# Safety and efficacy of MIKE-1 in patients with advanced pancreatic cancer: a study protocol for an open-label phase I/II investigator-initiated clinical trial based on a drug repositioning approach that reprograms the tumour stroma

**DOI:** 10.1186/s12885-022-09272-2

**Published:** 2022-02-24

**Authors:** Yasuyuki Mizutani, Tadashi Iida, Eizaburo Ohno, Takuya Ishikawa, Fumie Kinoshita, Yachiyo Kuwatsuka, Miwa Imai, Shinobu Shimizu, Toshihisa Tsuruta, Atsushi Enomoto, Hiroki Kawashima, Mitsuhiro Fujishiro

**Affiliations:** 1 Department of Gastroenterology and Hepatology, Nagoya University Graduate School of Medicine, Nagoya, Japan; 2 Department of Pathology, Nagoya University Graduate School of Medicine, Nagoya, Japan; 3 Department of Advanced Medicine, Nagoya University Hospital, Nagoya, Japan; 4 Department of Endoscopy, Nagoya University Hospital, 65 Tsurumai-Cho, Showa-Ku, Nagoya, 466-8550 Japan; 5 Department of Gastroenterology, Graduate School of Medicine, The University of Tokyo, Tokyo, Japan

**Keywords:** Cancer stroma, Tumour microenvironment, Cancer-associated fibroblasts, Cancer-restraining CAFs, Meflin, ISLR, AM80, Tamibarotene, MIKE-1

## Abstract

**Background:**

Cancer-associated fibroblasts (CAFs) are an important component of the tumour microenvironment. Recent studies revealed CAFs are heterogeneous and CAF subset(s) that suppress cancer progression (cancer-restraining CAFs [rCAFs]) must exist in addition to well-characterised cancer-promoting CAFs (pCAFs). However, the identity and specific markers of rCAFs are not yet reported. We recently identified Meflin as a specific marker of rCAFs in pancreatic and colon cancers. Our studies revealed that rCAFs may represent proliferating resident fibroblasts. Interestingly, a lineage tracing experiment showed Meflin-positive rCAFs differentiate into α-smooth muscle actin-positive and Meflin-negative CAFs, which are generally hypothesised as pCAFs, during cancer progression. Using a pharmacological approach, we identified AM80, a synthetic unnatural retinoid, as a reagent that effectively converts Meflin-negative pCAFs to Meflin-positive rCAFs. We aimed to investigate the efficacy of a combination of AM80 and gemcitabine (GEM) and nab-paclitaxel (nab-PTX) in patients with advanced pancreatic cancer.

**Methods:**

The phase I part is a 3 + 3 design, open-label, and dose-finding study. The dose-limiting toxicity (DLT) of these combination therapies would be evaluated for 4 weeks. After the DLT evaluation period, if no disease progression is noted based on the Response Evaluation Criteria in Solid Tumors (RECIST) version 1.1 or if the patient has no intolerable toxicity, administration of AM80 with GEM and nab-PTX would be continued for up to 24 weeks. The phase II part is an open-label, single-arm study. The maximum tolerated dose (MTD) of AM80 with GEM and nab-PTX, determined in phase I, would be administered until intolerable toxicity or disease progression occurs, up to a maximum of 24 weeks, to confirm efficacy and safety.

The primary endpoints are frequency of DLT and MTD of AM80 with GEM and nab-PTX in the phase I part and response rate based on the RECIST in the phase II part. Given the historical control data, we hope that the response rate will be over 23% in phase II.

**Discussion:**

Strategies to convert pCAFs into rCAFs have been developed in recent years. We hypothesised that AM80 would be a promising enhancer of chemosensitivity and drug distribution through CAF conversion in the stroma.

**Trial registration:**

Clinicaltrial.gov: NCT05064618, registered on 1 October 2021.

jRCT: jRCT2041210056, registered on 27 August 2021.

## Background

**Fig. 1:**
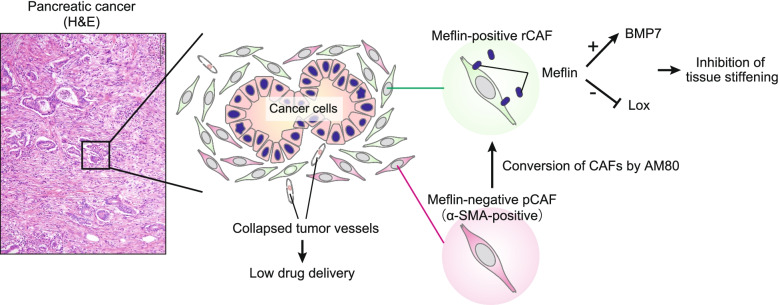
Representative histology of human pancreatic ductal adenocarcinoma (PDAC) and diversity of cancer-associated fibroblasts (CAFs). The representative histology of human PDAC is shown in the left panel. Note that the volume of the tumour stroma is predominant in cancer cells. In tumour stroma, there are many proliferating CAFs that deposit a large amount of the extracellular matrix. CAFs can be classified based on their functions into cancer-promoting CAFs and cancer-restraining (rCAFs). Meflin is a marker of rCAF and inhibits tissue fibrosis by augmenting bone morphogenetic protein 7 signalling and inhibiting lysyl oxidase activity. AM80 may induce the reprogramming of tumour stroma by upregulating the expression of Meflin in CAFs

Cancer-associated fibroblasts (CAFs) are a major component of tumour stroma, and many interventions to modulate or deplete CAFs have attempted to overcome the ‘stromal roadblock’ comprising CAFs and the extracellular matrix (ECM) produced by them [1–3]. CAF proliferation is observed in almost all types of cancer, and is most conspicuous in refractory cancers, such as pancreatic/bile duct cancers. In these cancers, the volume of stroma is often 5–10 times greater than that of tumour cells (Fig. 1).

CAFs produce a large amount of ECM that impedes the permeation of anticancer drugs, causing the resistance of tumours to anticancer drugs. Hence, with the expectation that treatment resistance can be improved by deleting cancer stroma, some research groups have attempted to suppress CAFs by inhibiting the Sonic hedgehog (Shh) pathway, which is crucial for CAF proliferation in preclinical models of pancreatic and bladder cancers [4, 5]. Another group examined the effect of genetic elimination of α-smooth muscle actin (SMA)-positive CAFs on tumour progression in an autochthonous pancreatic cancer mouse model [6]. All these attempts, however, revealed that neither the inhibition nor depletion of CAFs induces the regression of tumours; rather, they result in the progression and poor differentiation of the developed tumours. Furthermore, in a phase II study testing the efficacy of a combination of the anticancer drug gemcitabine (GEM) and a CAF inhibitor (Shh inhibitor IPI-926) in human patients with pancreatic ductal adenocarcinoma (PDAC), disease progression was observed following the administration of IPI-926 [7]. These studies have led to the notion that eliminating the stroma does not necessarily improve the outcome but rather aggravate the prognosis of pancreatic cancer. Another hypothesis was that either CAFs are tumour-suppressive, or there exist two types of CAFs—cancer-promoting CAFs (pCAFs) and cancer-restraining CAFs (rCAFs) (Fig. 1) [8]. However, despite the many previously reported pCAF markers, such as α-SMA, platelet-derived growth factor receptor, CXC chemokine ligand 12, podoplanin/aggrus, fibroblast activation protein 1, and rCAF-specific marker(s) remain unknown.

We recently identified Meflin, a glycosylphosphatidylinositol-anchored membrane molecule, as a functional maker of rCAFs in PDAC (Fig. 1) [9–12]. Meflin is specifically expressed in fibroblasts but not in other cell types across various tissues [13]. Recent studies have identified two molecules as candidates for proteins that interact with Meflin. One is fibrogenesis-suppressing cytokine bone morphogenetic protein 7 (BMP7) [14]. Meflin suppresses fibrogenesis by binding to BMP7 and potentiating its signal via its cognate receptor. Meflin also binds to lysyl oxidase (Lox), which regulates the cross-linking of collagen fibres and tissue induration to suppress its activity [15]. These findings suggest that the function of Meflin is to suppress fibrogenesis/tissue induration to keep tissues soft. Further studies on several preclinical pancreatic cancer models showed that Meflin expression confers the ability of CAFs to suppress tumour growth, leading to the hypothesis that Meflin is a marker of rCAFs in cancer.^9^ We further found via a lineage-tracing experiment that Meflin-positive rCAFs are converted into α-SMA-positive CAFs, which presumably represent pCAFs as the tumour grows.^9^ This indicates the existence of CAF plasticity between rCAFs and pCAFs, which may depend on the tumour microenvironment and context (Fig. 1).

A previous study showed that the administration of a vitamin D analogue to a pancreatic cancer mouse model induced changes in gene expression in CAFs, and this significantly improved tumour sensitivity to anticancer drugs.^3^ This phenotypic change of CAFs, which was termed ‘stromal reprogramming’, has attracted attention of researchers and pharmaceutical sectors as a new strategy to overcome stromal roadblock, leading to the initiation of several clinical trials that investigate the efficacy of combination therapies of vitamin D derivatives and anticancer drugs or immune checkpoint inhibitors in patients with unresectable advanced PDAC [16, 17].

We recently screened a library of ligands of nuclear receptors for reagents that induce the upregulation of Meflin expression using CAFs derived from human PDAC and identified AM580 as a reagent that induces nearly 20-fold stronger expression of Meflin than a vitamin D derivative or all-trans retinoid acid (ATRA), which is known to have an ability to reprogram cancer stroma, similar to vitamin D derivatives [15]. We also found that AM80 (general trade name: tamibarotene), which was developed as a structural isomer of AM580 and approved for acute promyelocytic leukaemia (APL) only in Japan, [18–20] also induced the expression of Meflin in CAFs to a similar extent as that of AM580 [15]. Certainly, oral administration of AM80 resulted in a decrease in α-SMA expression and an increase in Meflin expression in CAFs in a pancreatic cancer mouse model, suggesting that AM80 is capable of reprogramming pCAFs into rCAFs [15]. We next investigated the effect of the combination of AM80 and anticancer drugs (GEM and nab-PTX) in a xenograft mouse model of PDAC, which demonstrated that AM80 administration significantly improved the anti-tumour effect of GEM/nab-PTX without weight loss. AM80 monotherapy exerted no anti-tumour effects in this experiment. Consistent with the effects of Meflin on BMP7 signalling and Lox activity, AM80 administration induced a decrease in tumour stiffness and increase in tumour vessel area and intratumoral concentration of GEM. These effects were not observed in Meflin-deficient mice. Therefore, it was suggested that AM80 might potentiate the effect of anticancer drugs by increasing the number of Meflin-positive rCAFs or reprogramming pCAFs into rCAFs. Although hypothetical, the data also suggested the possibility that AM80 administration increases in tumour vessel area and drug delivery were ascribed to a decrease in interstitial pressure resulting from ameliorating tissue fibrosis.

The data from the preclinical studies described above suggest that AM80 may also enhance the efficacy of conventional chemotherapeutics in patients with PDAC. The present study aimed to investigate the efficacy of the combination therapy of AM80 (developmental code: MIKE-1) and GEM and nab-PTX in patients with unresectable PDAC.

## Methods/design

### Patient selection

- Patients with untreated PDAC incapable of curative resection (stage III or IV) will be included.
- Patients with remote metastasis/unresectable locally advanced PDAC will be included.
- Patients with postoperative recurrence or borderline resectable PDAC will not be included.

### Inclusion criteria

Patients with unresectable pancreatic cancer histologically or cytologically diagnosed with adenocarcinoma based on the Classification of Pancreatic Carcinoma 4th English Edition published by the Japan Pancreas Society [21] who meet the following criteria:

- Patients who have previously had no anticancer treatment for the disease (radiotherapy, chemotherapy, immunotherapy, surgery, or study treatment)
- Patients not younger than 20 years and not older than 79 years at the time of obtaining informed consent
- Patients with one or more measurable lesions found in the primary foci of the pancreas on contrast-enhanced computed tomography (CT) at screening based on Response Evaluation Criteria in Solid Tumors (RECIST) version 1.1
- Patients expected to survive for 12 weeks or more after the start of the study
- Patients capable of understanding the details of the study and submitting written consent
- Patients with Eastern Cooperative Oncology Group Performance Status of 0 or 1
- Patients who meet the following criteria in the blood test within 7 days before enrolment and have organ functions maintained (if transfusion has been performed, the test should be performed after a 2-week interval or later):
  Total bilirubin ≤ institutional upper limit of normal (ULN) × 1.5 (≤ 3.0 mg/dL in cases undergoing biliary drainage)
  Aspartate transaminase AST (glutamic oxaloacetic transaminase) and alanine aminotransferase (glutamic pyruvic transaminase) ≤ ULN × 3 (≤ ULN × 5 if abnormal liver function is present due to a malignant tumour)
  Creatinine ≤ 1.5 mg/dL or creatinine clearance ≥60 mL/min (if actual creatinine clearance is unavailable, use the estimated value)
  White blood cell count ≥ 3,500/mm^3^ and ≤ 12,000/mm^3^
  Neutrophil count ≥ 1,500/mm^3^
  Platelet count ≥ 100,000/mm^3^
  Haemoglobin ≥ 9.0 g/dL
  Prothrombin activity ≥ 70%
- Patients capable of undergoing treatment in an outpatient setting
- Patients capable of swallowing oral drugs and continuing the medication
- For women with childbearing potential, patients capable of preventing pregnancy for 30 days before the start of the investigational dosing, during the study period, and for at least 2 years after the end of the study.
- Patients capable of undergoing biopsy from the pancreatic tumour within 28 days before and 8 weeks after the start of the study treatment (Day 57: acceptable range ± 7 days)

### Exclusion criteria

- Patients who meet any of the following criteria will be excluded from the study:
  Patients with poorly controlled heart disorder (congestive cardiac failure, myocardial infarction/angina pectoris unstable within 1 year before enrolment, or arrhythmia requiring treatment)
  Patients with diabetes mellitus with inadequate control or hypertension
  Patients with active autoimmune disease requiring systemic steroid or immunosuppressive therapy
  Patients with infection requiring systemic antimicrobials or antivirals
  Patients with interstitial pneumonia or pulmonary fibrosis (currently ≥ Grade 2)
- Patients receiving any other investigational drugs or products (except for existing chemotherapy agents or placebo) within 4 weeks before enrolment
- Patients with confirmed brain metastasis (if brain metastasis symptoms are present, confirmed by head CT or magnetic resonance imaging)
- Patients with ascites or pleural effusion requiring drainage
- Patients meeting one of the following conditions:
  Hepatitis B antigen positive
  Hepatitis C virus (HCV) antibody positive and HCV-RNA positive
  Human immunodeficiency virus antibody positive
- Patients with peripheral sensory or motor neuropathy ≥ Grade 2
- Patients with double cancer (double cancer refers to synchronous double cancer and heterochronic double cancer with disease-free survival within 5 years, excluding carcinoma in situ assessed as being cured by local treatment or a lesion equivalent to intramucosal carcinoma)
- Patients undergoing surgery within 4 weeks before enrolment (excluding diagnostic biopsy or staging laparoscopy)
- Patients with bleeding tendency or coagulation abnormalities that prevent safe biopsy under endoscopic ultrasound (e.g., history or complications of serious intratumoral bleeding, coagulation abnormality, or haemorrhagic disorder)
- Patients with history of allergy to the investigational drug, the concomitant chemotherapy, any of their additives, or vitamin A product
- Patients requiring anticoagulants
- Patients with cerebral infarction, pulmonary infarction, other arterial or venous thrombosis, or their sequelae
- Patients with gastrointestinal disease that may affect absorption of the investigational drug
- Pregnant or breastfeeding female patients (except for those who discontinue and do not resume breastfeeding)
- Male patients with a female partner who wants to be pregnant
- Patients with hypervitaminosis A
- Patients under vitamin A medication or using a supplement containing vitamin A on a usual basis (enrolment is acceptable if the administration is discontinued at consent submission)
- Patients who are judged to be unsuitable by the investigator

## Study treatment

**Fig. 2:**
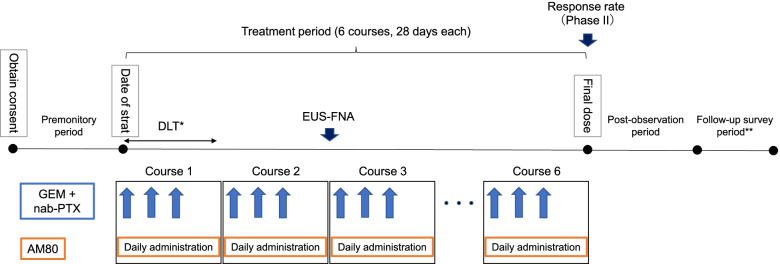
Protocol of the present study. In the present study, AM80 will be administered daily for 4 weeks and repeated for up to six courses. Each course consists of gemcitabine (GEM) (1,000 mg/m^2^) and nab-paclitaxel (nab-PTX) (125 mg/m^2^) administered intravenously over 30 min on Days 1, 8, and 15 without administration at Week 4. After completing Course 6, if no disease progression is noted based on the Response Evaluation Criteria in Solid Tumors version 1.1 or if the patient has no intolerable toxicity, GEM + nab-PTX will be continued as a usual treatment. In such a case, continuous GEM/nab-PTX will be considered as post-treatment. ^*^Dose-limiting toxicity will be evaluated in a 4-week period only in the phase I trial. ^**^The follow-up period will be set in all cases until the date of completion of the post-observation period (cut-off) in all cases

This study is a phase I/II open-label study in patients with unresectable PDAC (Fig. 2, Table 1).

**Table 1:**
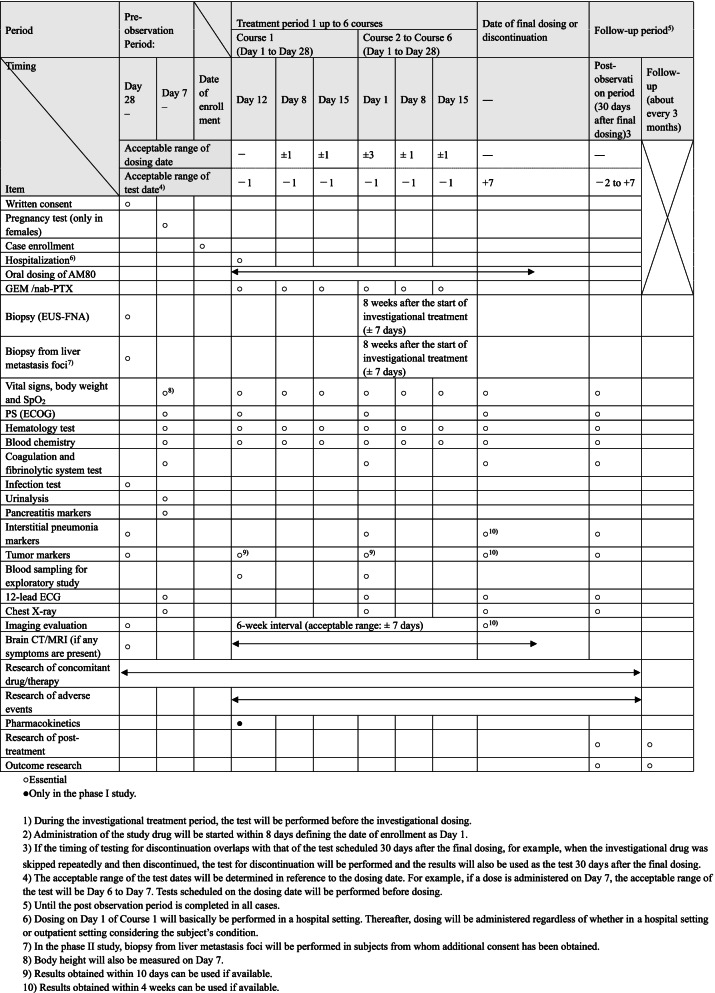
Items and timing of observation, tests and research

○Essential

●Only in the phase I study

1) During the investigational treatment period, the test will be performed before the investigational dosing.

2) Administration of the study drug will be started within 8 days defining the date of enrollment as Day 1.

3) If the timing of testing for discontinuation overlaps with that of the test scheduled 30 days after the final dosing, for example, when the investigational drug was skipped repeatedly and then discontinued, the test for discontinuation will be performed and the results will also be used as the test 30 days after the final dosing.

4) The acceptable range of the test dates will be determined in reference to the dosing date. For example, if a dose is administered on Day 7, the acceptable range of the test will be Day 6 to Day 7. Tests scheduled on the dosing date will be performed before dosing.

5) Until the post observation period is completed in all cases.

6) Dosing on Day 1 of Course 1 will basically be performed in a hospital setting. Thereafter, dosing will be administered regardless of whether in a hospital setting or outpatient setting considering the subject’s condition.

7) In the phase II study, biopsy from liver metastasis foci will be performed in subjects from whom additional consent has been obtained.

8) Body height will also be measured on Day 7.

9) Results obtained within 10 days can be used if available.

10) Results obtained within 4 weeks can be used if available.

**Fig. 3:**
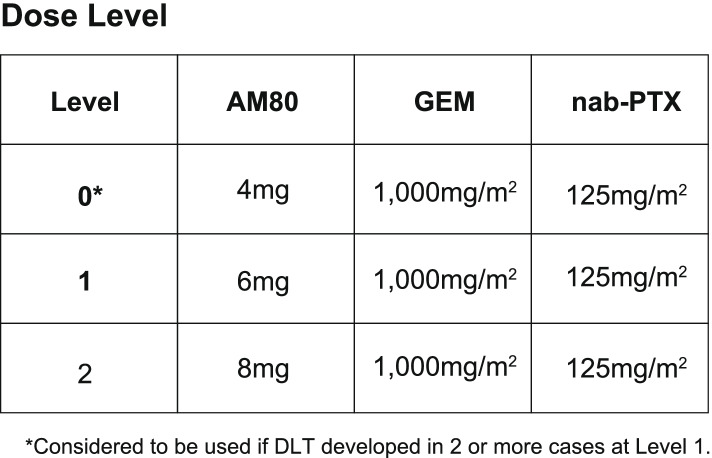
Dose escalation in the phase I part. The AM80 dose will not be reduced or escalated in the same subject. Each course consists of gemcitabine (1,000 mg/m^2^) and nab-paclitaxel (125 mg/m^2^) administered intravenously over 30 min on Days 1, 8, and 15, without administration at Week 4. The course will be repeated, and the dose will be reduced ad libitum based on the subject’s condition in compliance with the criteria of this protocol

**Fig. 4:**
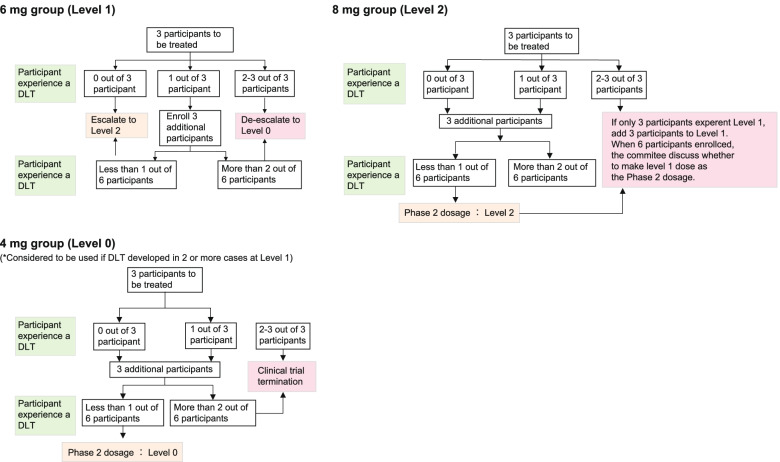
Flowcharts of steps to determine dose-limiting toxicities in the phase I study. In the phase 1 part study, this is a dose-escalation study using a standard 3 + 3 design. See text for details

Phase I part: The phase I part of the study is a dose-escalation study using a standard 3 + 3 design. Three cases will constitute one cohort, and the dose will gradually be adjusted according to the number of cases with the onset of DLT in Course 1 (Figs. 2, 3, and 4). A pharmacokinetics study will also be performed **(**Table 2).

**Table 2:** Schedule of pharmacokinetic study (Start day of the AM80 dosing in the phase I study)

Day of measurement	Day of dosing (Day 1)						Day 2
Follow-up (hr)	0	1	2	4	8	10	24
Dosing of investigational drug	○						
Blood sampling	○^a^	○	○	○	○	○	○^a^

Pharmacokinetic study on the start day of the AM80 dosing. The acceptable range of blood sampling time 1, 2, 4, 8, 10 and 24 h after dosing: the range will be about 15 min before and after the scheduled time after 1, 2, and 4 h and within 30 min before and after the scheduled time after 8 h

Pre-dosing blood will be sampled after breakfast and then the drug will be administered orally. Then, GEM/nab-PTX and premedication drugs will be administered in the methods usually performed in the institution. ^a^Blood will be sampled before taking the drug

Phase II part: The clinically recommended dose determined in the phase I study will be used (Fig. 2, Table 1).

### Design of the phase I part and definition of dose-limiting toxicity

- The onset of DLTs will be evaluated after AM80 dosing for each subject in phase I.
- The DLT evaluation period is until Day 28, defining the date of the start of the investigational drug as Day 1. The severity of adverse events will be assessed by an investigator based on CTCAE version 5.0.
- DLT is defined as an event that falls under any of the following items among the adverse events that develop during the above DLT evaluation period and is possibly related to the AM80 and GEM/nab-PTX combination therapy:
  i Grade 4 haematotoxicity persisting for more than 7 days
  ii Non-haematological toxicity ≥ Grade 3 persisting for more than 7 days, even if symptomatically treated
  iii An adverse event that impedes the administration of GEM or nab-PTX on Days 8 and 15 in Course 1
  iv An adverse event that impedes the administration of GEM or nab-PTX on Day 8, leading to dose reduction on Day 15 in Course 1
- As haematopoietic growth factor products, such as granulocyte colony-stimulating factor, may cause underestimation of DLT, its use as primary prevention during the DLT evaluation period is prohibited.
- The investigator will accumulate potential DLTs in every three cases (as DLT evaluation cases) and determine whether they are DLTs with advice from members of the Efficacy and Safety Evaluation Board. The investigator will decide the addition of cases to the same dose level, the movement to the next dose level, or discontinuation of the entire study according to the incidence status of DLTs based on evaluation results from the Efficacy and Safety Evaluation Board.

### Design of the phase II part

- This is an open-label study conducted to assess the efficacy and safety of AM80 with GEM and nab-PTX.
- The AM80 dose will be fixed at the clinically recommended dose determined in phase I and administered orally twice daily after breakfast and dinner for consecutive days. The treatment will be continued until the development of intolerable toxicity or disease progression or up to six courses to confirm efficacy and safety.
- The final evaluation will be conducted in a total of 43 cases.

### Definition of course

- AM80 will be administered orally daily for up to six courses, each of which consists of 4 weeks.
- AM80 will be used concomitantly with GEM and nab-PTX and can be continued until any toxicity intolerable for the patient develops, disease progression is confirmed based on RECIST version 1.1, and/or administration is discontinued at the discretion of the investigator or the patient’s request within the six courses.
- Each course consists of GEM (1,000 mg/m^2^) and nab-PTX (125 mg/m^2^) administered intravenously over 30 min on Days 1, 8, and 15 without administration at Week 4. The course will be repeated, and the dose will be reduced ad libitum based on the subject’s condition in compliance with the criteria of this protocol.
- Each course will consist of 28 days, regardless of whether AM80 is withdrawn or GEM/nab-PTX dosing is skipped/postponed.
- For both AM80 and GEM and nab-PTX, Day 1 of each course will be Day 28 + 1 of the previous course from Course 2. Initial dosing of GEM and nab-PTX can be postponed to 3 weeks after the same day of the week. AM80 can be withdrawn until 8 weeks, the same day of the week after at the latest. The start of the AM80 course will conform to Day 1 of GEM and nab-PTX in Course X.
- Tests and observations required to judge the start, postponement, dose reduction, and discontinuation of GEM and nab-PTX will be performed between the day before the dosing and the time before the dosing.
- After completing Course 6, if no disease progression is noted based on RECIST version 1.1 or if the patient has no intolerable toxicity, GEM + nab-PTX will be continued as a usual treatment. In such a case, continuous GEM and nab-PTX will be considered as post-treatment.
- If AM80 ends at Course 6, the case will move to the post-observation period.

### Pharmacokinetics

A pharmacokinetics study will be performed on the day of the start of the investigational dosing in the phase I study (Table 2).

## Endpoints

### Primary endpoints

- Phase I part: DLT
  The number of DLT cases noted within the period between the start of treatment and Day 28 and their incidence (%) will be calculated by the level.
- Phase II part: response rate

Response rate is defined as the rate calculated with the number of cases analysed as the denominator and the number of patients with the best overall response assessed as complete response (CR) or partial response (PR) as the numerator based on RECIST version 1.1.

### Secondary endpoints

- Overall survival
  Overall survival is defined as the period from the date of the start of investigational dosing to the date of death for any reason, defining the date of completion of the post-observation period in all cases as the cut-off.
- Progression-free survival
  Progression-free survival is defined as the period from the date of the start of investigational dosing to the date when progression is identified or date of death if the subject dies without identifying progression (regardless of cause), defining the date of completion of the post-observation period in all cases as the cut-off.
- Blood MIKE-1 concentration
  Plasma MIKE-1 concentration will be confirmed at each time point (Table 2).
- Response rate (in phase I)

Response rate will be evaluated similar to phase II study response rate (RR).

### Safety endpoints

- Incidence (%) of adverse events
- Incidence (%) of serious adverse events
  The development of adverse events during the study period will be evaluated. Adverse reactions and serious adverse events will also be evaluated.
- Vital signs and laboratory values

Vital signs and laboratory values will be reviewed at the time points specified in the Schedule of Assessments.

## Statistical analysis

### Phase I part

We will calculate the incidence of DLT and 95% confidence interval (CI) from the start of the protocol treatment to the end of the first course for each dose level. Clopper-Pearson method will be used to calculate 95% confidence interval.

### Phase II part

The RR and 90% CI will be calculated. The Clopper–Pearson method will be used to calculate the 90% CI.

## Sample size

### Phase I part

- Three or six cases at each dose level
- As the major purpose of the phase I study is to confirm the safety (tolerability) of this combination therapy to study MTD, the sample size will not be designed based on statistical rationale.

### Phase II part

- Forty-three cases
- As GEM and nab-PTX combination therapy resulted in CR rate < 1% and PR rate of 23% in a previous study [22], the null hypothesis of the RR is assumed to be less than or equal to 23%. Although there has been no previous study on the add-on effect of concomitant AM80, the clinically expected effect is assumed to be approximately 20%, and the expected RR is set to be 43% [22]. Assuming a two-sided significance level of 10% and a power of 80%, 38 cases will be required. Estimating the dropout rate to be approximately 10%, the target number of cases has been set to 43.

## Data monitoring

Monitoring of the study will be performed by the Nagoya University Research Center.

## Discussion

The use of AM80 as the sole regimen resulted in good outcomes in patients with APL with a total CR rate of 61.5%, but in the phase I/II clinical trial for hepatocellular carcinoma conducted from 2009 to 2014, the CR rate was 0% (0/25), and PR and stable disease were observed in 1/25 cases and 7/25 cases, respectively. The disease control ratio was 32% (95% CI, 15.0–53.5), which did not meet the initial goal [23]. Thus, it has been suggested that AM80 hardly exerts its anti-tumour effects on solid tumours. The concept of our present study is different from those of previous studies in that we utilise AM80 as a drug that induces reprogramming of the tumour microenvironment but does not target cancer cells as described above.

Notably, a recent phase I study that tested the efficacy of ATRA plus GEM/nab-PTX in patients with PDAC showed a favourable RR in 48% of the patients [24]. Given that AM80 is a synthetic retinoid that exhibits significantly higher stability to light, heat, and oxidation than ATRA, we expect that our present study will also lead to a positive outcome. Another advantage of AM80 is its high tolerability in patients with APL.

Finally, given that CAF proliferation with fibroinflammatory reactions is found across many types of human cancers, we expect that AM80-medicated reprogramming of tumour stroma could be applied to many types of refractory cancers in the future.

## Data Availability

Not applicable.

## Abbreviations

GEM: Gemcitabine
ATRA: All-trans retinoic acid
PTX: Paclitaxel
PDAC: Pancreatic ductal adenocarcinoma
APL: Acute promyelocytic leukaemia
DLT: Dose-limiting toxicity
CR: Complete response
PR: Partial response
